# Supplementary material for: Viewing low back pain through the lens of spinal evolution: Understanding the morphology and limits of the human spine
Source: PLoS One. 2026 Jan 16;21(1):e0339032. doi: 10.1371/journal.pone.0339032 (PMC12810782; doi:10.1371/journal.pone.0339032)
Supplement: S1 File — All data underlying the findings reported in this study are fully available within the article. No additional datasets, images, or external repositories were used. (DOCX) [file pone.0339032.s001.docx]

**Location of the data (i.e. the repository name)**

The addresses of all data used in our scientific study are provided below.

**Figure 1 A-F**:

References:

1A: User: Clemens Schmillen. Title: Rock paintings from the Cave of Beasts (Gilf Kebir, Libyan Desert) Estimated 7000 BP. (Accessed: 13.02.2024, <https://commons.wikimedia.org/wiki/File:Bestias11.JPG>)

1B: User: Alessandro Passare, Title: Round Head figures and zoomorphic figures, including a Barbary sheep (Accessed: 13.02.2024, <https://commons.wikimedia.org/wiki/File:Fondazione_Passar%C3%A9_V1_056.jpg>)

1C: User: Bernard Gagnon, Title: Rock Shelters of Bhimbetka (Accessed: 12.02.2024, <https://commons.wikimedia.org/wiki/File:Rock_Shelter_8,_Bhimbetka_02.jpg>)

1D: User: Jimbfleak, Title: San rock paintings from the Western Cape in South Africa. (Accessed: 13.02.2024, <https://commons.wikimedia.org/wiki/File:Southafrica468bushman.jpg>)

1E: User: Ricardo Andre Frantz, Title: Cave painting at Serra da Capivara National Park, Brazil. (Accessed: 10.02.204, <https://commons.wikimedia.org/wiki/File:Serra_da_Capivara_-_Several_Paintings_2b.jpg>)

1F: User: Laca Galuzzi Title: Rock paintings in Tadrart Acacus region of Libya dated from 12,000 BC to 100 AD. (Accessed: 09.02.2024, <https://en.m.wikipedia.org/wiki/File:Libya_4924_Pictograms_Tadrart_Acacus_Luca_Galuzzi_2007_cropped.jpg>

**Figure 2 A-F**:

**References 2A**: Artist: Norman de Garis Davies Title: Craftsmen, Tomb of Nebamun and Ipuky, User: Pharos Accessed: 05.02.2024, <https://commons.wikimedia.org/wiki/File:Craftsmen,_Tomb_of_Nebamun_and_Ipuky_MET_eg30.4.103a.jpg>

**2B**: Author: Photographed by the British Museum; original artist unknown Date: Photograph published 2001; artwork created c. 1300 BC User: [A. Parrot](https://commons.wikimedia.org/wiki/User:A._Parrot) Accessed: 01.03.2025, <https://tr.wikipedia.org/wiki/M%C4%B1s%C4%B1r#/media/Dosya:BD_Weighing_of_the_Heart.jpg>

**2C:** Author: [Olaf Tausch](https://commons.wikimedia.org/wiki/User:Oltau) Date: 16 October 2019 Source: Own work User: [Neoclassicism and Enthusiast](https://commons.wikimedia.org/wiki/User:Neoclassicism_Enthusiast) Accessed: 01.03.2025, <https://upload.wikimedia.org/wikipedia/commons/f/f7/Kairo_Museum_Statuette_Cheops_03_%28cropped%29.jpg>

**2D:** Author**:** [Olaf Tausch](https://commons.wikimedia.org/wiki/User:Oltau), Date: 16 October 2019, Source: Own work, User: [Oltau](https://commons.wikimedia.org/wiki/User:Oltau), Accessed: 01.03.2025, <https://upload.wikimedia.org/wikipedia/commons/a/a8/Kairo_Museum_Sitzstatue_Chephren_06.jpg>

**2E**: Author: [Djehouty](https://commons.wikimedia.org/wiki/User:Djehouty), Date: 29 March 2016, Source: Own work, User: [Djehouty](https://commons.wikimedia.org/wiki/User:Djehouty) Accessed: 01.03.2025, <https://upload.wikimedia.org/wikipedia/commons/4/41/%C3%84gyptisches_Museum_Kairo_2016-03-29_Rahotep_Nofret_01.jpg>

**2F**: Author: Daniel Csörföly, Date: 2007 January-February, Source: Photos taken by [Daniel Csörföly](https://commons.wikimedia.org/wiki/User:Cs%C3%B6rf%C3%B6ly_D), User: [Csörföly and D](https://commons.wikimedia.org/wiki/User:Cs%C3%B6rf%C3%B6ly_D) Accessed: 01.03.2025, https://upload.wikimedia.org/wikipedia/commons/2/26/Aswan%2C_Egypt_WestBankTombs_2007jan15._14_byDanielCsorfoly.JPG

**Figure 3 A-F**:

.

**3 A**: Title: The Harvesters Artists: Pieter Brueghel the Elder, 1565. User: Rolf Kranz, (This work is in the public domain in its country of origin and other countries and areas where the copyright term is the author's life plus 100 years or fewer.) (This work is in the public domain in the United States because it was published (or registered with the U.S. Copyright Office) before January 1, 1929.) Accessed: 13.02.2024, <https://commons.wikimedia.org/wiki/File:Pieter_Bruegel_the_Elder-_The_Harvesters_-_Google_Art_Project.jpg>

**3 B**: Title: The Red Vineyard / Red Vineyard at Arles. Artist: Vincent van Gogh. User: Coldcreation. This work is in the public domain in its country of origin and other countries and areas where the copyright term is the author's life plus 100 years or fewer. Accessed: 10.02.2024, <https://commons.wikimedia.org/wiki/File:Red_vineyards.jpg>

**3C**: Title: A Maid Milking a Cow in a Barn, Artist: Gerard ter Borch, c. 1652-54, User: [Onderwijsgek](https://commons.wikimedia.org/wiki/User:Onderwijsgek) , Accessed: 1.03.2025, <https://upload.wikimedia.org/wikipedia/commons/e/e5/Gerard_ter_Borch_%28II%29_%22De_Koestal%22.jpg>

**3D**: Title: Potato Planters, Artist: [Jean-François Millet](https://en.wikipedia.org/wiki/en:Jean-Fran%C3%A7ois_Millet), User: [DcoetzeeBot](https://commons.wikimedia.org/wiki/User:DcoetzeeBot), Accessed: 1.03.2025, <https://upload.wikimedia.org/wikipedia/commons/9/9d/Jean-Fran%C3%A7ois_Millet_-_Potato_Planters_-_Google_Art_Project.jpg>

**3E**: Title: Monthly calendar of tasks, Artist: [Master of the Geneva Boccaccio](https://www.wikidata.org/wiki/Q3303311), User: [Il and Dottore](https://commons.wikimedia.org/wiki/User:Il_Dottore), Date: 1470 – 1475, <https://upload.wikimedia.org/wikipedia/commons/1/1b/Crescenzi_calendar.jpg>

**3F**: Title: Breezing Up (A Fair Wind), Artist: [Winslow Homer](https://en.wikipedia.org/wiki/en:Winslow_Homer) , Date: between 1873 and 1876, User: [Trzęsacz](https://commons.wikimedia.org/wiki/User:Trz%C4%99sacz), Accessed: 1.03.2025, https://upload.wikimedia.org/wikipedia/commons/d/d7/Winslow_Homer_-_Breezing_Up_%28A_Fair_Wind%29.jpg

**Figure 4 A-E**:

References: **4A**: Title: *Army Post Office 3, Boulogne, 1919. Artists:* [John Lavery](https://en.wikipedia.org/wiki/en:John_Lavery)  (1856–1941) Users: [Rcbutcher](https://en.wikipedia.org/wiki/User:Rcbutcher). This work is in the [public domain](https://en.wikipedia.org/wiki/public_domain) in its country of origin and other countries and areas where the [copyright term](https://en.wikipedia.org/wiki/List_of_countries%27_copyright_lengths) is the author's life plus 70 years or fewer. This work is in the [public domain](https://en.wikipedia.org/wiki/public_domain) in the [United States](https://en.wikipedia.org/wiki/United_States) because it was [published](https://commons.wikimedia.org/wiki/Commons:Publication) (or registered with the [U.S. Copyright Office](https://en.wikipedia.org/wiki/United_States_Copyright_Office)) before January 1, 1929 Accessed: [27.11.2024], <https://upload.wikimedia.org/wikipedia/commons/4/4a/Army_Post_Office_3%2C_Boulogne%2C_1919_by_John_Lavery.jpg>

**4B:** Title: A Day at a Leeds Flax Mill, Artists: No picture credit in book, Date: 1919, original image from 1843, Users: [Tagishsimon](https://commons.wikimedia.org/wiki/User:Tagishsimon), Accessed: 04.03.2025, <https://upload.wikimedia.org/wikipedia/commons/6/6c/Marshall%27s_flax-mill%2C_Holbeck%2C_Leeds_-_interior_-_c.1800.jpg>

**4C:** Description: Missouri Governor [Joseph W. Folk](https://en.wikipedia.org/wiki/Joseph_W._Folk) inspecting [child laborers](https://en.wikipedia.org/wiki/child_labor), 1906, drawn by [Marguerite Martyn](https://en.wikipedia.org/wiki/Marguerite_Martyn) of the *St. Louis Post-Dispatch, Date:* 29 April 1906, Source: Original publication: "St. Louis Post-Dispatch, April 29, 1906, User: [BeenAroundAWhile](https://commons.wikimedia.org/w/index.php?title=User:BeenAroundAWhile&action=edit&redlink=1), Accessed: 04.03.2025 <https://upload.wikimedia.org/wikipedia/commons/b/b1/Missouri_Governor_Joseph_Folk_inspecting_child_laborers%2C_1906%2C_drawn_by_Marguerite_Martyn.jpg>

**4D**: Title: The casting of iron in blocks, Artists: [Herman Heijenbrock](https://en.wikipedia.org/wiki/en:Herman_Heijenbrock)  (1871–1948), User: [Qlama9](https://commons.wikimedia.org/w/index.php?title=User:Qlama9&action=edit&redlink=1)  Accessed: 04.03.2025 <https://upload.wikimedia.org/wikipedia/commons/f/f0/1890heyenbrock.jpg>

**4E:** Description: From www.victorianweb.org/history/ashley.html, a educational site offering free info on the victorian age. Image is a copy of one from an official report of a parliamentary commission done in the mid 19th century, Date: [18:41, 11 October 2007](https://upload.wikimedia.org/wikipedia/commons/7/7b/Coaltub.png), User: [Skies](https://commons.wikimedia.org/w/index.php?title=User:Skies&action=edit&redlink=1) , Accessed: 04.03.2025 https://upload.wikimedia.org/wikipedia/commons/7/7b/Coaltub.png

**Figure 5 A-E**:

Reference: **5A:** Title: Painting of factory workers Artist: Toni Anton Wolter. User: Wmpearl (This work was never published prior to January 1, 2003, and is currently in the public domain in the United States) Accessed: 13.02.2024, <https://commons.wikimedia.org/wiki/File:Painting_of_factory_workers_by_Toni_Anton_Wolter.jpg>

**5B: Title:** Annual, 1899 (May), Date: 1899-05; 1899, User: [Ssafder](https://commons.wikimedia.org/w/index.php?title=User:Ssafder&action=edit&redlink=1) , Accessed: 13.02.2024 <https://upload.wikimedia.org/wikipedia/commons/f/f5/Annual%2C_1899_%28May%29_-_DPLA_-_14ca89cfa3717f287f4fd0a902420297_%28page_54%29_%28cropped%29.jpg>

**5C:** Description: Steam engine technology, Date: 1894, Source: Brockhaus' Konversations-Lexikon, 14.Auflage, 4.Band, Author: F.A. Brockhaus, Berlin und Wien, Permission: Author died more than 70 years ago - public domain, User: [Hgrobe](https://commons.wikimedia.org/wiki/User:Hgrobe), Accessed: 04.03.2025, <https://upload.wikimedia.org/wikipedia/commons/7/73/Dampfhammer2_brockhaus.jpg>

**5D**: Author: [William Armstrong](https://en.wikipedia.org/wiki/en:William_Armstrong_(Canadian_artist))  (1822–1914), Description: English: Painting of the Toronto Rolling Mills, an iron rails factory founded in 1857 by a group of businessmen led by railway magnate Sir Casimir Gzowski. At that time, it was the largest iron mill in Canada and the largest manufacturer in Toronto. The introduction of steel rails led to its closure in 1873., Date: 1864, Source/Photographer: This image is available from the [Toronto Public Library](https://en.wikipedia.org/wiki/Toronto_Public_Library) under the reference number JRR 1059, User: [Skeezix1000](https://commons.wikimedia.org/wiki/User:Skeezix1000), Accessed: 04.03.2025, <https://commons.wikimedia.org/wiki/File:Toronto_Rolling_Mills.jpg>

**5E:** Description: English: [Croton Aqueduct](https://en.wikipedia.org/wiki/Croton_Aqueduct) shutoff valve, illustration in [Harper's Weekly](https://en.wikipedia.org/wiki/Harper%27s_Weekly) magazine, November 12, 1881, Date: Issue date: November 12, 1881; uploaded May 1, 2008, Author: W. St. John Hasker[?] (difficult to read artist's name (at bottom right — in caption), User: [Beyond My Ken](https://commons.wikimedia.org/wiki/User:Beyond_My_Ken), Accessed: 04.03.2025, https://upload.wikimedia.org/wikipedia/commons/d/da/HarpersWeeklyIllustrShuttingOffTheCroton11121881_crop.jpg

**Figure 6 A-E**:

Reference: **6A:** Title: The Munitions Girls Artist: Stanhope Alexander Forbes) User: Fæ This file is licensed under the Creative Commons Attribution 4.0 International license. Accessed: 16.02.2024 <https://en.m.wikipedia.org/wiki/File:%27The_Munitions_Girls%27_oil_painting,_England,_1918_Wellcome_L0059548.jpg>

**6B: Title:** Woman Spinning, Artist: [Jean-François Millet](https://en.wikipedia.org/wiki/en:Jean-Fran%C3%A7ois_Millet), Date: 1855-186 User: [Trzęsacz](https://commons.wikimedia.org/wiki/User:Trz%C4%99sacz), Accessed: 04.03.2025, https://commons.wikimedia.org/wiki/File:Jean-Fran%C3%A7ois_Millet_-_Femme_filature_(1855-60).jpg

**6C:** Title: The Stonebreakers, Artist: [Gustave Courbet](https://en.wikipedia.org/wiki/en:Gustave_Courbet)  (1819–1877), Date: 1849, User: [JarektUploadBot](https://en.wikipedia.org/wiki/User:JarektUploadBot), Accessed: 04.03.2025, <https://en.wikipedia.org/wiki/File:Gustave_Courbet_-_The_Stonebreakers_-_WGA05457.jpg> **6D**: Description: Gargantua, a lithography by Honoré Daumier, Date: 16 December 1831, Author: [Honoré Daumier](https://en.wikipedia.org/wiki/en:Honor%C3%A9_Daumier)  (1808–1879), User: [Racconish](https://commons.wikimedia.org/wiki/User:Racconish), Accessed: 04.03.2025, <https://commons.wikimedia.org/wiki/File:Honor%C3%A9_Daumier_-_Gargantua.jpg>

**6E**: Title: A Cotton Office in New Orleans, Artist: [Edgar Degas](https://en.wikipedia.org/wiki/en:Edgar_Degas)  (1834–1917), Date: 1873, User: [SreeBot](https://commons.wikimedia.org/wiki/User:SreeBot) , Accessed: 04.03.2025, https://commons.wikimedia.org/wiki/File:Cottonexchange1873-Degas.jpg
