# Supplementary material for: Viewing low back pain through the lens of spinal evolution: Understanding the morphology and limits of the human spine
Source: PLoS One. 2026 Jan 16;21(1):e0339032. doi: 10.1371/journal.pone.0339032 (PMC12810782; doi:10.1371/journal.pone.0339032)
Supplement: Attachment — Submitted filename: PLOS ONE_Art Science_review_9_18_2025.docx [file pone.0339032.s002.docx]

This is a novel and qualitative piece of work that blends science and art to explore how evolution may have influenced the spine and contributed to the progression of back pain in the human race.

Please find my minor comments below:

- **Page 4, second paragraph:** The letter **‘T’** is missing in the sentence beginning *“hese profound societal changes coincided with increased spinal strain, repetitive tasks, and urbanization, all contributing to a discernible rise in spinal and musculoskeletal disorders [9-11].”*
- **Methods section:** Please add figure numbers to the text for clarity, as it is currently difficult to determine which figure is being referenced.
- **Discussion section:** Some words are unnecessarily bolded; this formatting should be removed.
