# Supplementary material for: Viewing low back pain through the lens of spinal evolution: Understanding the morphology and limits of the human spine
Source: PLoS One. 2026 Jan 16;21(1):e0339032. doi: 10.1371/journal.pone.0339032 (PMC12810782; doi:10.1371/journal.pone.0339032)
Supplement: Attachment — Submitted filename: Scientist 2.docx [file pone.0339032.s004.docx]

**Dear Scientist 2**

We would like to express our sincere gratitude for your thoughtful and constructive feedback, which has significantly enhanced the quality, clarity, and scientific depth of our manuscript. Your insightful comments offered valuable perspectives that guided us in refining both the structure and the interpretation of our findings.

Throughout this process, we have learned a great deal from your expertise and critical insight, which has been truly enriching for us as researchers. Your guidance not only improved the precision of our arguments but also inspired us to approach the topic from a more comprehensive and balanced perspective.

We deeply appreciate the time and effort you devoted to reviewing our work and for helping us bring this study to a higher academic standard.

With warmest regards and sincere appreciation,
Abdullah Emre Taçyıldız *on behalf of all co-authors*

**Reviewer Comment 1**

Fascial and Myofascial Chain Perspective Consider referencing Thomas W. Myers’ “Anatomy Trains” model or Stecco’s fascial continuity research, which demonstrate how dysfunctions in one segment (e.g., thoracolumbar fascia, multifidus, quadratus lumborum) propagate tension throughout the kinetic chain. Chronic seated or flexed postures typically cause shortening of the hip flexors and thoracolumbar fascia, inhibition of the multifidus, and compensatory overactivity of the quadratus lumborum and upper trapezius—mechanically linking the pelvis, sacroiliac joint (SIJ), and shoulder girdle dysfunctions.

**Response to Reviewer’s Comment 1:**

As suggested, we have now clarified the functional integration of the spine within the global myofascial network by adding the following paragraph to the revised manuscript:

“Recent myofascial research, including the ‘Anatomy Trains’ model proposed by Myers and the fascial continuity studies of Stecco, emphasizes that the spine functions as part of an integrated kinetic chain [33–36]. Dysfunction in one segment—such as thoracolumbar fascial shortening or multifidus inhibition—may propagate tension through interconnected myofascial pathways, leading to compensatory overload in the pelvis, sacroiliac joint, and shoulder girdle [34–36]. These mechanisms reinforce our hypothesis that modern postural habits generate systemic biomechanical stress exceeding the spine’s evolutionary design (Figure 1–6).”

This paragraph has been incorporated into the revised version to address the reviewer’s suggestion and to strengthen the theoretical framework of the manuscript.

**Reviewer Comment 2**

Trigger Points and Postural Overload Introduce the role of myofascial trigger points (TrPs) as a micro-level consequence of prolonged overload or static posture. Citing Travell & Simons could strengthen the pathophysiological basis: sustained seated posture → ischemic muscle zones → TrP activation in the multifidus and quadratus lumborum → referred pain mimicking spinal disorders. The addition of ultrasound (US)-based confirmation of TrPs and US-guided dry needling (US-DN) (Bubnov R., Ultrasound Med Biol, 2011; https://link.springer.com/article/10.1186/1878-5085-3-13) would provide a modern pathophysiological bridge between evolutionary mismatch and clinical manifestations of low back pain.

**Response to Reviewer’s Comment 2**
In response to the reviewer’s valuable suggestion, we have expanded the discussion of the micro-physiological mechanisms underlying postural overload. The following paragraph has been added to the revised manuscript:

“At a micro-physiological level, prolonged static or flexed postures induce ischemic and hypoxic zones within postural muscles such as the multifidus and quadratus lumborum, facilitating myofascial trigger point (TrP) formation [37]. These TrPs can produce referred pain patterns closely resembling spinal pathology, thereby blurring the clinical distinction between functional and structural disorders. Recent ultrasound-based studies further confirm the presence of TrPs and demonstrate the therapeutic role of US-guided dry needling (US-DN) in reducing neuromyofascial hyperactivity [38, 39]. Together, these findings provide a mechanistic bridge linking evolutionary postural mismatch to the clinical manifestations of chronic low back pain (Figure 1–6).”

This paragraph was incorporated to strengthen the physiological and clinical correlation between modern postural behavior and spinal evolution, as recommended.

**Reviewer Comment 3:**

Comparative Functional Anatomy
The authors could contrast human static sitting posture with that of non-human primates (e.g., chimpanzees), which maintain a flexed hip and mobile lumbar spine position even when seated (see <https://doi.org/10.1016/j.applanim.2021.105417>; <https://doi.org/10.1016/0163-6383(91)90022-K>). This comparison would show that humans’ prolonged sitting with posterior pelvic tilt and loss of lumbar lordosis is not “evolutionarily optimized,” unlike dynamic squat resting seen in apes.

**Response to Reviewer’s Comment 3:**
To further strengthen the comparative anatomical perspective as suggested, we have added the following paragraph to the revised manuscript:

“From a comparative anatomical standpoint, non-human primates such as chimpanzees maintain a flexed hip and dynamically mobile lumbar spine even when seated, preserving segmental motion and balanced load transmission across the pelvis and spine [40–43]. In contrast, the modern human sitting posture—marked by posterior pelvic tilt, reduced lumbar lordosis, and static hip flexion—deviates from these adaptive resting behaviors. This suggests that human prolonged sitting is not an evolutionarily optimized posture but rather a biomechanically compromised one emerging from cultural and occupational adaptations.”

This addition reinforces the evolutionary framework of the manuscript by contrasting human postural mechanics with those of non-human primates.

### ****Reviewer Comment 4:****

The authors might briefly acknowledge modern behavioral adaptations—such as the slightly head-down posture that improves social perception but may alter cervical–thoracic load distribution (see https://oa.mg/work/10.1016/j.actpsy.2022.103602)—to highlight ongoing tension between evolutionary optimization and modern psychosocial demands.

**Response to Reviewer’s Comment 4:**

In accordance with the reviewer’s insightful comment, we have enriched the discussion by integrating recent behavioral perspectives that link modern social habits to postural adaptation. The following paragraph has been added to the revised version:

“Recent behavioral studies indicate that modern humans often adopt a slightly head-down posture to enhance social perception and empathy cues in interpersonal communication [44]. While this adaptation may serve psychosocial purposes, it simultaneously increases cervical and upper thoracic mechanical load, illustrating the continuing conflict between evolutionary optimization and the postural demands of modern social behavior.”

This addition provides a behavioral and psychosocial dimension to the discussion, emphasizing how contemporary communication patterns may contribute to cervical and thoracic strain within an evolutionary context.
