# Supplementary material for: Viewing low back pain through the lens of spinal evolution: Understanding the morphology and limits of the human spine
Source: PLoS One. 2026 Jan 16;21(1):e0339032. doi: 10.1371/journal.pone.0339032 (PMC12810782; doi:10.1371/journal.pone.0339032)
Supplement: Attachment — Submitted filename: Scientist response.docx [file pone.0339032.s005.docx]

**Dear Scientist and Reviewer**

We would like to sincerely thank the reviewer not only for helping us improve the overall quality and clarity of our manuscript, but also for providing constructive insights from which we have learned a great deal scientifically. Their thoughtful and formative feedback has significantly enriched both the intellectual depth and the scientific rigor of our work.

**Reviewer Comment 1**: Conceptual Balance

While the manuscript argues that civilization and modern posture negatively affect spinal health, the examples introduced (e.g., primate posture, chimpanzee lumbar mobility, and cultural balancing practices such as head-load carrying in African populations) actually demonstrate that posture adaptation is multifactorial and not always detrimental. This complexity slightly contradicts the paper’s main narrative and should be acknowledged briefly in the discussion.

**Author response 1:**

We sincerely thank the reviewer for this insightful comment highlighting the need for conceptual balance. We fully agree that postural evolution is not a unidirectional process of degeneration but rather a multifactorial adaptation shaped by evolutionary, environmental, and cultural influences.

Accordingly, we have **revised the relevant section** of the discussion to clarify that the cited examples—such as habitual head-load carrying in African populations and lumbar flexibility in non-human primates—do not contradict our central argument. Instead, they **contextualize modern spinal vulnerability** as a mismatch between our evolved spinal morphology and the rapid biomechanical and behavioral changes brought by modern civilization.

This conceptual refinement strengthens the manuscript by integrating the reviewer’s valuable perspective and emphasizing that spinal health reflects both adaptive and maladaptive postural mechanisms operating along an evolutionary continuum. The following paragraph has been added accordingly.

While our central argument emphasizes that modern civilization has introduced postural challenges through sedentary behavior, artificial ergonomics, and diminished proprioceptive feedback, it is equally important to recognize that postural evolution itself is not a unidirectional process of degeneration. Anthropological and biomechanical research shows that posture has historically been a multifactorial adaptive trait shaped by environmental and cultural demands. For instance, studies of traditional African populations (Luo tribe) practicing habitual head-load carrying demonstrated **reduced energetic cost and highly efficient load transfer** during gait, reflecting adaptive strengthening of spinal and paraspinal structures [55, 56]. Women of the Kikuyu tribe, in contrast, carry substantial loads supported by a forehead strap, a practice that frequently leads to the development of a permanent cranial groove [55]. Likewise, comparative anatomical analyses indicate that non-human primates, including chimpanzees, exhibit **distinct lumbar and pelvic adaptations** that provide context-specific mobility and axial function, underscoring that spinal form varies according to ecological pressures [6]. These findings refine our argument by showing that posture has always adapted to its mechanical environment; thus, modern civilization may represent a **mismatch** between our evolved spinal design and the novel loading patterns of contemporary life. In this view, the issue is not evolutionary failure, but the rapid divergence between ancestral biomechanical demands and present-day postural exposures.

**Reviewer Comment** 2. Functional and Clinical Integration

To strengthen translational value, the authors could still expand on the functional anatomy aspects—particularly the role of the thoracolumbar fascia, multifidus, quadratus lumborum, and pelvic chain—and how chronic overload leads to myofascial dysfunction. Reference to treatments based on understanding biomechanics via myofascial trigger points, ultrasound diagnostics, and US-guided dry needling (US-DN) would help illustrate how the evolutionary–postural concept translates into clinical understanding and intervention.

**Author Response 2:**

In response to the reviewer’s valuable suggestion to enhance the translational relevance of the manuscript, we expanded the discussion on functional anatomy—specifically addressing the biomechanical roles of the thoracolumbar fascia, multifidus, quadratus lumborum, and the pelvic myofascial chain—and clarified how chronic asymmetric loading contributes to myofascial dysfunction. In addition, we integrated a brief overview of clinically relevant interventions grounded in biomechanical understanding, including the significance of myofascial trigger points, the role of ultrasound-based diagnostic approaches, and the therapeutic use of ultrasound-guided dry needling (US-DN). These revisions aim to more clearly bridge the evolutionary–postural framework with practical clinical applications. The following paragraph has been added accordingly.

Ultrasound (US) imaging has increasingly become a critical tool in the diagnosis and treatment of myofascial pain. **Bubnov** first demonstrated the feasibility of *real-time sonographic visualization* of myofascial trigger points and reported **93.3% pain relief** in a clinical series of 91 patients treated with US-guided trigger-point dry needling (US-DN) [42]. Building on these findings, a **comparative clinical study** showed that US-guided needling provides **more accurate localization**, **greater pain reduction**, and **higher treatment responsiveness** than palpation-guided techniques [41]. More recent reports further suggest that US-guided interventions may enhance **muscle function** and **regional mobility** by enabling targeted treatment of fascial dysfunction [43]. Collectively, these data underscore how image-guided approaches translate myofascial biomechanics into precise clinical interventions, reinforcing the connection between fascial anatomy, postural evolution, and modern rehabilitation strategies. These ultrasound-guided myofascial findings support our central hypothesis by showing that the fascial and segmental dysfunctions produced by modern, evolution-mismatched loading patterns are not only measurable but clinically reversible. Thus, US-based interventions provide mechanistic confirmation that contemporary postural strain exceeds the adaptive conditions under which the human spine evolved.

From a practical standpoint, the evolutionary–postural framework highlights the need for interventions that restore dynamic spinal loading and myofascial mobility. Approaches such as dynamic posture training, fascial stretching, and core stabilization can counteract the sedentary, asymmetric loading patterns characteristic of modern environments [36]. In addition, ultrasound-guided dry needling (US-DN) provides a targeted method for reducing fascial densification and improving segmental control [40, 41]. Together, these strategies translate evolutionary principles into clinically actionable rehabilitation pathways.

**Reviewer Comment** 3: Practical Perspective

The discussion would benefit from a short preventive and rehabilitative outlook, referring to dynamic posture training, fascial stretching, or core stabilization, US-DN, to connect evolutionary theory with clinical application.

**Author Response 3:**

In accordance with the reviewer’s insightful recommendation to incorporate a concise preventive and rehabilitative perspective, we expanded the discussion to highlight clinically relevant strategies such as dynamic posture training, fascial stretching, core stabilization, and the complementary use of ultrasound-guided dry needling (US-DN). These additions were made to strengthen the bridge between the evolutionary framework and practical clinical management. The paragraph below has been added to the revised manuscript to address this point.

From a functional and clinical standpoint, the evolution of human posture cannot be fully understood without integrating the role of the myofascial system. The thoracolumbar fascia (TLF) forms a continuous tension network linking the spine, pelvis, and lower limbs, providing mechanical stability and efficient force transmission during movement [36, 37]. Within the evolutionary context of human bipedalism, the thoracolumbar fascia (TLF) functions as a key connective interface linking the trunk and pelvis, contributing to load transfer along the axial chain and stabilizing the spine during upright locomotion. In this framework, Schleip et al. further demonstrated that fascia is not a passive membrane but a contractile tissue: human lumbar fascia contains α-SMA–positive myofibroblasts capable of generating active tension, and subtle stiffness changes in these fascial layers may influence neuromuscular coordination through mechanosensory pathways, thereby integrating with the broader biomechanical adaptations that support human spinal posture [38]. Together, these findings support our central hypothesis that modern postural problems stem less from skeletal alignment alone and more from maladaptive changes within the myofascial system. If the thoracolumbar fascia and its paraspinal–pelvic networks function as contractile, load-responsive tissues prone to densification, stiffness shifts, and neuromuscular modulation, then the sedentary and asymmetric loading patterns of contemporary life may disrupt a system that once stabilized the trunk and coordinated lower-limb biomechanics. In this sense, modern postural dysfunction reflects an evolutionary mismatch between a formerly dynamic fascial architecture and today’s mechanically constrained environments.
